# The Assessment of Toxicity Characteristics of Cellular Uptake of Paramagnetic Nanoparticles as a New Magnetic Resonance Imaging Contrast Agent

**DOI:** 10.22037/ijpr.2019.1100823

**Published:** 2019

**Authors:** Hamidreza Moghimi, Reza Zohdiaghdam, Nader Riahialam, Zhaleh Behrouzkia

**Affiliations:** a *Department of Pharmaceutics and Pharmaceutical Nanotechnology, School of Pharmacy, Shahid Beheshti University of Medical Sciences, Tehran, Iran. *; b *Departmentof Medical Imaging School of ParaMedicine, Urmia University of Medical Sciences, Urmia, Iran.*; c *Department of Medical Physics, School of Medicine, Tehran University Medical Sciences, Tehran, Iran. *; d *Department of Medical Physics, School of Medicine, Urmia University Medical Science, Urmia, Iran.*

**Keywords:** Nanoparticles, Cytotoxicity, Cell line, Gd2O3-DEG, Magnetoliposomes

## Abstract

Nanoparticles are unique that enable many promising medical and technological applications in their physical, and chemical properties. It is widely accepted that nanoparticles should be thoroughly tested for health nanotoxicity, but a moderate risk analysis is currently prevented by a revealing absence of mechanistic knowledge of nanoparticle toxicity. The purpose of this study was to assess *in-vitro* cytotoxicity of Gadolinium oxide with diethylene glycol polymer (Gd_2_O_3_-DEG) and magneto liposome nanoparticles (MLNs) in Hepa 1-6 cell lines as models to assess nanotoxicity *in-vitro*. The effects of magnetic nanoparticles on these cell lines were evaluated by light microscopy and standard cytotoxicity assays. The underlying interactions of these nanoparticles with physiological fluids are key characteristics of the perception of their biological efficacy, and these interactions can perhaps be performed to relieve unpleasant toxic effects. Our results demonstrated that the Gd_2_O_3_-DEG and MLNs had significantlydifferent non-cytotoxic effects. Our results suggest that these cell lines provide valuable models to assess the cytotoxicity of nanoparticles *in-vitro*. The results of the present study demonstrated that MLNs and Gd_2_O_3_-DEG with lower longitudinal relaxation time (T_1_) than Gadolinium Pentetic acid (Gd-DTPA) in Hepa 1-6 cell lines are sensitive positive Magnetic Resonance Imaging (MRI) contrast agents that could be as attractive as candidates for cellular and molecular lipid content targets such as liver diagnostic applications. These data reveal that MLNs is a useful positive contrast agent for targeting and cell tracking. This will help to image of cells and special organs like liver that uptakes liposomal formulation very well.

## Introduction

Liposomes as carriers for controlled delivery of drugs that have been drawing much attention which is because of encapsulation of many substances in aqueous and lipid phases (1). Despite the useful properties of nanoparticles, their side effects, mechanism, and their toxicity should be investigated. The important fact about nanoparticles is their remarkable reactivity due to their size, surface chemistry, and oxidative stress. These characteristics may result in toxic effects (2).

The small sizes of nanoparticles increase their surface area and more chemical molecules which attach this surface and increase its reactivity or toxic effects. In addition, these small size nonmaterials pass through cell membranes especially into the mitochondria (2, 3). Despite beneficial characteristics of nano sized particles for drug delivery and contrast agents, they could result unwanted side effects. Both the surface chemistry and chemical components of nanoparticles are the surfaces assumed as important factors, so that the surface modification of nanoparticles can reduce toxicity. The reduction of the toxicity of drugs and contrast agents such as iron oxide or paramagnetic nanoparticles have been shown by coating them with different coatings (3).

Recently, nonmaterial’s such as Gd_2_O_3_, super paramagnetic nanoparticles (SPIO) with/without polymeric coating have received enormous attention for their ability to create new types of contrast agents in magnetic resonance imaging (4). Gadolinium is a paramagnetic substance in its trivalent state and is used for MRI because of its strong effect on decreasing the T_1 _relaxation times of the tissues. However, gadolinium shows the potential toxicity due to its seven unpaired electrons and therefore could be decreased by chelating and coating. The primary magnetic properties of diethylene glycol (DEG) of this group were reported, as a new surface covering material in combination with Gd_2_O_3_ nanoparticles (5). However, the synthesis procedure and effective size and agglomeration of gadolinium nanoparticles coated with DEG materials should be studied more (6-8). For this reason, these two groups of surface coating materials (DEG and liposome) even could be useful for covering nanoparticles in biomedical cellular and molecular imaging applications.

For example, cancer cells are more flexible towards nanoparticle toxicity than normal cells due to an increased rate of proliferation and metabolic activity (9, 10). The differences between toxic effects are even observed for nanoparticles of the same materials. The main objective of this study was to evaluate toxicity on biological structures such as biological cells. Therefore, selection of the appropriate cell type that is based on target introduction methods of nanomaterials is an important factor in cytotoxicity assays. For this purpose, Gd_2_O_3_-DEG was prepared in new synthetic controlled method and cytotoxicity assays.

This study would be involved in nanoparticles, which are the composition of Gd_2_O_3_, DEG, and liposome. Gd_2_O_3_ nanoparticles with Diethylene Glycol Polymer could produce a good MR signal and therefore could be a useful potential contrast medium for cell tracking in molecular magnetic resonance imaging (mMRI). In this study, Gd_2_O_3_ nanoparticles were used as a model of nonmaterial in order to evaluation of *in-vitro* potential toxicity in Hepa 1-6 cell lines.

## Experimental

*Preparation of Gd2O3-DEG*

Gd_2_O_3_-DEG was prepared through the polyol method, previously reported by this group (11). Next, as a part of the new method, the colloidal liquid was cooled, centrifuged, filtered at 2000 rpm for 0.5 h by using 0.2 µm filter (polyethersulfone, Viva science Sartorius, Hannover, Germany). Free Gd^3+^ ions and excess DEG in the solution were eliminated by a 1000 MW membrane (Dialysis tubing, benzoylated, Sigma, USA) for 24 h. The agglomerated nanoparticles were removed by 12000 Da membrane (Dialysis tubing cellulose membrane, Sigma, USA) for 24 h across distilled water.

*Preparation of Magnetoliposomes*

Liposomes were prepared by lipid-film hydration method (6). Distearoylphosphatidylcholine: Cholesterol (DSPC:Chol) (DSPC, Lipoid GmbH, Germany) (Chol, Sigma, Germany) with a molar ratio 50:50. A mixture of the appropriate amounts of lipids (typically: 50 mmols of total lipid) was dissolved in chloroform/methanol 2:1 (v/v) and evaporated for 2 h in order to dry by rotary evaporation at 65 °C. Then, appropriate amounts of Gd_2_O_3_-DEG in distilled water were added to lipid film and hydrated for 2 h. After preparation of the Magnetoliposomes (ML), sonication for 0.5 h and dialysis for 24 h were performed in order to remove unentrapped Gd_2_O_3_-DEG (7). *In-vitro* dilutions with five different concentration forms 0.3, 0.6, 0.9, 1.2, 1.5 mM of Gd-DTPA, Gd_2_O_3_-DEG, and MLNs were prepared.

*The Characterization Study*

The concentration of the Gd_2_O_3_-DEG and ML nanoparticles was determined by inducing coupled plasma-atomic (ICP) (AES-Varian-Liberty 150 AX Turbo-USA), and after paramageto liposomes nanoparticles (PMLNs) sonication, dialysis of the membrane was performed. The measurements of particle morphology and hydrodynamic diameter were performed by transmission of electron microscopy (TEM) (Philips, Model CM 120, Netherland) and dynamic light scattering (DLS), (Malvern Instrument-UK). 

*Cell Culture*

Even though these results may not accurately predict the *in-vivo* toxicity, it provides a basis for understanding the mechanism of toxicity and nanoparticle uptake at the cellular level (12). The Hepa 1-6 was derived from BW7756 tumor in a C57L mouse (National Cell Bank of Iran, Pasteur Institute of Iran). The cells were cultured in DMEM (Invitrogen, Auckland, New Zealand) with 2 mM glutamine and 2 g/L sodium bicarbonate (Sigma, St Louis, MO) adjusted to contain 4.5 g/L glucose and 10% fetal bovine serum (FBS) (Invitrogen) and 1% penicillin/streptomycin (Invitrogen, Carlsbad, CA) at 37 °C in a 5% CO_2_ atmosphere.

*Microculture tetrazolium test (MTT assay)*

The inhibitory effect of Gd-DTPA, Gd_2_O_3_–DEG nanoparticles, and PMLNs on the growth and proliferation of Hepa 1-6 cell lines were assessed by up-taking of thiazolyl blue tetrazolium bromide (MTT, Sigma) by viable cells (13). The cells were plated onto 96-well plates (Orange Scientific, Brussels, Belgium) at a density of 1.0 × 10^4^ cell/100 µL/well. After incubation at 37 °C for 24 h, the medium was replaced with either control medium or medium containing specialized concentration Gd_2_O_3_ for 24, 48 and 72 h. The concentration of Gd_2_O_3_ for cell culture treatment was 0.3, 0.6, 0.9, 1.2 mM, respectively and the concentration of zero means negative control. One-hundred microliter of MTT solution (0.5 mg/mL) was added to each well and then the cells incubated at 37 °C for 3 h. Following solubilize the precipitated formazan with 100 µL DMSO, the optical densitometry was measured at a wave length of 570 nm. The inhibition rate (IR) was evaluated using the following equation: IR (%) = 1 - OD_exp_/OD_con _× 100, where OD_exp_ and OD_con_ are the optical densitometries of treated and untreated cells, respectively. The viability rate of Gd_2_O_3_ was evaluated using the following equation: Viability (%) = 100 - IR (%) (14). 

*LDH leakage*

LDH assay was performed for detecting the cytotoxicity index of Gd_2_O_3_-DEG nanoparticles Gd-DTPA and Magnetoliposomse nanoparticles in the cell medium by using a commercially available kit (Roche Applied Science). 

Serum Lactate Dehydrogenase (LDH) Assay*—*Serum stored at 20 °C was used for this assay, which was performed according to the manufacturer’s protocol (Roche Applied Science) (15). Briefly, after the exposure of Hepa 1-6 cell lines to Gd_2_O_3_ -DEG nanoparticles, Gd-DTPA and PMLNS for 2 h, 100 µL of supernatant (cell medium+ Serum+ Nanoparticles) was transferred into an optically clear 96-well flat-bottomed microtiter plate. The concentration of Gd_2_O_3_ for cell culture treatment was 0.3, 0.6, 0.9, 1.2, 1.5 mM, respectively and the concentration of zero means negative control. To determine LDH activity, 100 µL of the reaction mixture was added to each well and incubated for 30 min at 15–25 °C. The control group is 0.1% DMSO (Dimethyl sulfoxide). The spectrophotometer was calibrated to zero absorbance using culture medium without cells. The relative LDH leakage (%) related to control wells containing cell culture medium without nanoparticles or PBS as a vehicle was calculated by (A)_test_/(A)_control _× 100.

The mark (A)_test_ is the absorbance of the test sample and (A)_control_ is the absorbance of the control sample. The absorbance of the samples was measured at 490 nm using an ELISA reader. Released LDH in culture supernatants causes the conversion of a tetrazolium salt (INT) into a red formazan product. The amount of color formed is proportional to the number of lysed cells. Cytotoxicity is expressed relative to the released basal LDH by untreated control cells (16).

*Statistical Analysis*

Statistical analysis was done by using repeated measures ANOVA for comparing paired samples (within-subjects) and One-way ANOVA for between subjects (compared means between groups). *P-*values less than 0.05 were considered statistically significant. The percentages of cell viability were presented graphically in the form of histograms, using Microsoft Excel computer program. Data are expressed as mean ± standard deviation (SD). All experiments were examined in triplicate. 

## Results

*Characterization of the contrast materials*

Table 1 shows the size and polydispersity index (PdI) measurements using the DLS, thereby, Gd_2_O_3_-DEG and PML nanoparticles had a hydrodynamic diameter distribution of 27.29 ± 6.10 nm with a PdI of 0.357, and the size of 128.86 ± 36.24 nm with the PdI of 0.387, respectively.

The results showed that with increasing the molecular weight, the nanoparticles size increased as well. However, despite their different sizes, PdI’s nanoparticles (as an index of the nanoparticles dispersion) had acceptable ranges of less than 0.5. 

**Figure 1:**
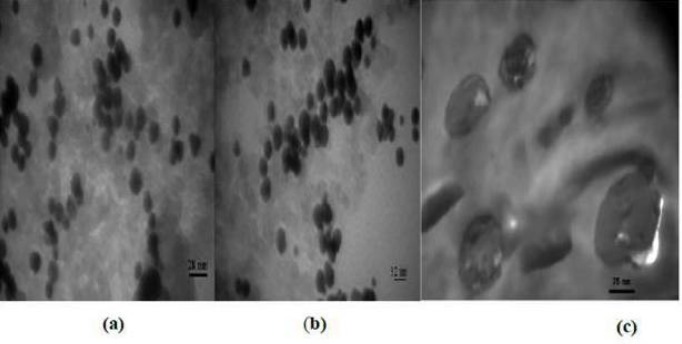
TEM images of nanoparticles. TEM images for (a, b) Gd2O3-DEG (c) Magnetoliposomes: uniformity and spherical or ellipsoidal shaped for Gd2O3-DEG and agglomeration for two other nanoparticles are observed

Figure 1 shows the morphology of three wrapped around nanoparticles, while specifically, just images of Gd2O3-DEG are sharp and uniformly such that spherical or ellipsoidal shape of Gd nanomagnetic particles could be visualized separately with clear grains in nano dimensions. Whiles the images of magneto liposomes could not be viewed as sharp as Gd_2_O_3_-DEG nanoparticles among their surface covers because of large molecular weights were agglomerated such that.

*Cell Morphology*

**Figure 2:**
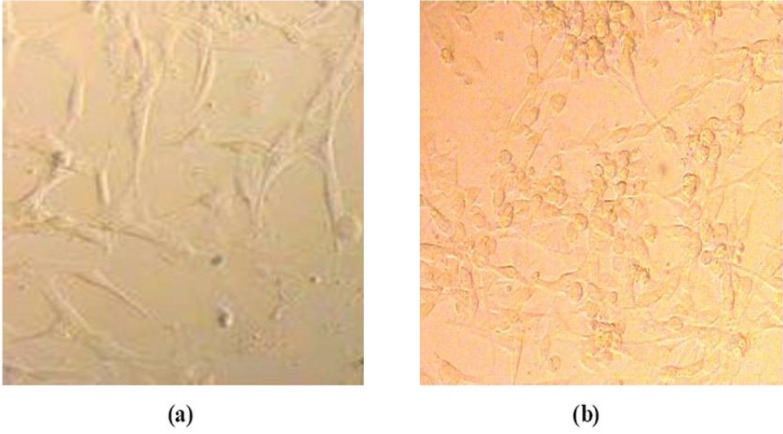
Morphological characterization of the cells. Cells were treated with different concentrations of different nanoparticles in DMEM and incubated for 24 h at 37 °C in a 5% CO2 atmosphere. Hepa 1-6 cell lines after incubation with Gd2O3-DEG for 24 h. (a) Hepa 1-6 cell lines control (b) Hepa 1-6 cell lines after incubation with Gd2O3-DEG for 24 h

The general morphology of the cell incubated with nanoparticles in phase-contrast microscopy is shown in Figure 2. At the end of 24 h, the exposed cells were washed with PBS and the cells were visualized by phase-contrast microscopy.

Figure 2 shows Hepa 1-6 cell lines that were well spread after incubation with Gd_2_O_3_-DEG for 24 h, and there was no distinct change in morphology after incubation for 24 h with any concentration of Gd_2_O_3_-DEG nanoparticles relatives. Similar results were obtained with Magneto liposomes.

*Microculture tetrazolium test (MTT assay)*

**Figure 3:**
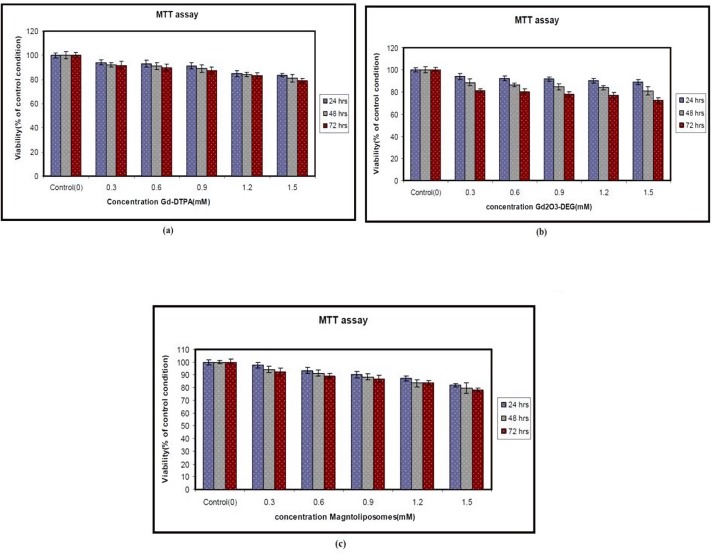
Effect of nanoparticles and Gd-DTPA on metabolic function in Hepa 1-6 cells. (a) Gd-DTPA, (b) Gd2O3-DEG, (c) Magnetoliposomes. The data are expressed as mean ± SD of three independent experiments

The Metabolic function of the cells was measured by means of the MTT assay after culturing in the presence of the nanoparticles for 24, 48, and 72 h. As is evident from Figure 2, the Gd_2_O_3_ nanoparticles with DEG, Magnetoliposomes had no signiﬁcant effect. Hepa 1-6 cell lines were exposed to different doses of nanoparticles for 24, 48, and 72 h as indicated in the Materials and Methods. Each point represents a mean value and standard deviation of 3 experiments. Cell Viability in different concentrations is not significantly different (*P < *0.05) compared to the control, as is evident from Figure 3. The MTT assay showed that Gd_2_O_3_-DEG nanoparticles are more toxic than other nanoparticles.

*LDH leakage*

The cellular toxicity of gadolinium oxide nanoparticles *in-vitro *was determined by measuring the releasing of the enzyme lactate dehydrogenize (LDH) into the cell culture supernatants with 24, 48, and 72 h incubation period and the gadolinium concentration of all particles. In order to control completeness of LDH releasing, one sample was lysed and its LDH value was taken as 100% toxicity. The effect was comparable to the exposure to 0.1% (v/v) DMSO. 

Concentration-dependent effects of mM on cytotoxicity of Hepa 1-6 cell line. The cells were treated with designated concentrations of mM and 0.1% (v/v) of DMSO as a vehicle control. 

After 72 h, LDH leakage was assayed in supernatants and cytotoxicity was expressed as a LDH% release. Statistically, different values of *P *= 0.005 (0.6 mM), *P *= 0.013 (0.9 mM), *P *= 0.023 (1.2 mM), and *P *= 0.003 (1.5 mM) were determined for 72 h after plating, compared to the control.

Since the LDH assay detects that lysis and cells arenot viable, the cells need to be destroyed for these investigations to optimize test conditions. Therefore, the positive control Triton X-100 was used, at the concentration of 2% to complete lysis of cells. 

**Figure 4:**
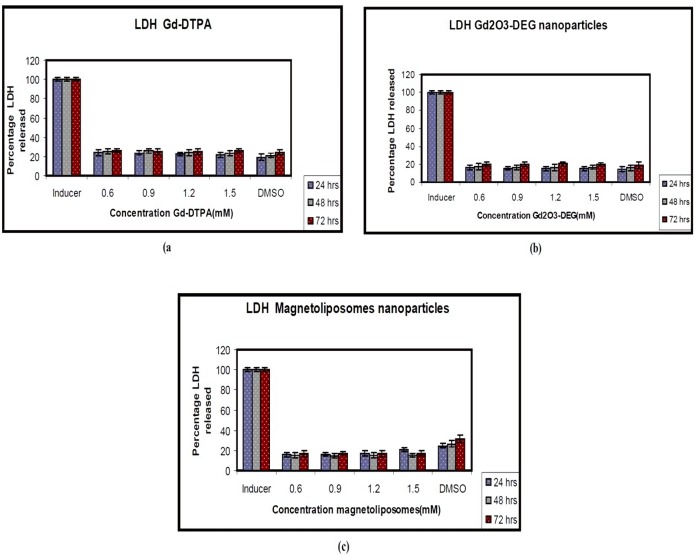
Toxicity of the Gd-DTPA and nanoparticles as measured by the release of the cytosolic enzyme lactate dehydrogenase (LDH) to Hepa 1-6 cells. DMSO (0) as control test (a) Gd-DTPA (b) Gd2O3-DEG (c) Magnetoliposomes. This figure shows that soluble Gd O - DEG and Magnetoliposomes have no effect on the plasma membrane at any of the concentrations tested. The data are expressed2 a3s mean ± SD of three independent experiments

The results of the LDH assay showed Gd-DTPA and two nanoparticles did not produce cytotoxicity in different concentrations. There were no significant differences between two nanoparticles and Gd-DTPA is used as commercially contrast agents in MRI department (Figure 4). 

There was a slight increase in LDH leakage at different doses of Gd_2_O_3_-DEG compared to other nanoparticles but Gd-DTPA was not statistically significant (*P *< 0.05). However, the results were similar for MTT and LDH assay (Figures 3 and 4). 

## Discussion

Many harmful effects of nanoparticles could be imagined at the cellular level by interacting with vital cell components such as membrane and mitochondria. The physicochemical properties of nanoparticles can affect their pharmacokinetics such as distribution, metabolism, and absorption.

Contrast agents can modify the signal intensity in different tissues by enhancing their contrast and also improving the low sensitivity of magnetic resonance imaging. Recently, studies have shown high efficiency and sensitivity of contrast agents when they have been used in the form of nanoparticles. For having higher relaxivity, reducing toxicity, increasing biocompatibility and half-life, besides preventing the nanoparticles aggregations, contrast agents should be coated with various materials in MRI. 

The development of suitable molecular magnetic resonance imaging (mMRI) by using contrast agents, is very important (17). Many methodical reports have been manifested in the last decade, which highlighted this issue, with the goal of understanding the interactions between different types of nanoparticles and cells as functions of size, shape, and surface chemistry of the nanomaterials (18).* In-vitro *studies using different cell systems demonstrated varying grades of prion inflammatory and oxidative-stress–related cellular answers after doing with laboratory-generated or filter collected environment ultrafine particles (19, 20). Gadolinium with seven unpaired electrons and gadolinium oxide are used as contrast agents for magnetic resonance imaging (MRI). Despite good relaxivity, Gd has high toxicity and it might be chelated or conjugated with other materials such as polymers or liposomes. Some primary magnetic properties of diethylene glycol (DEG) in combination with Gd oxide has been reported previously as a new surface covering material. On the other hand, because of its considerable physicochemical properties, liposome has drawn notable interest incovering the nanoparticles surfaces (21-25). For these reasons, these two groups of surface conjugated materials (DEG and Liposomes) could be useful for covering nanoparticles in biomedical cellular and molecular imaging applications. For this purpose, Gd_2_O_3_-DEG was prepared in new synthetic controlled method. However, little is known about how these coatings influence the toxicity of these particles. Therefore, we compared toxicity of contrast agent nanoparticles with Gd_2_O_3_ core and two different coatings with conventional contrast agent Gd-DTPA. As these contrast agents might be used for detection of Liver cancer, Hepa 1-6 cell lines were selected as a convenient *in-vitro* model to assess toxicity. Selection of a suitable cytotoxicity assay method is also an important consideration, since some may interfere with the actual toxic effect produced by the nanoparticle (26-29). For toxicity evaluations, cellular morphology, mitochondrial function (MTT assay), membrane leakage of lactate dehydrogenase (LDH assay) were assessed under control and exposed conditions (24 h, 48 h, 72 h of exposure).

Figure 2 shows the morphology of Gd_2_O_3_-DEG nanoparticle-exposed cells. Despite the relatively higher toxicity (LDH and MTT assay) in comparison to other nanoparticles and Gd-DTPA, no relevant differences were observed in for the different concentration of Gd_2_O_3_-DEG.

The results demonstrated that exposure to Gd based nanoparticles for 24 h resulted in a concentration-dependent increase in LDH leakage and exhibited is significant (*P *< 0.05) in cytotoxicity of different concentrations. Cell viability in different concentrations is not significantly different (*P < *0.05) in comparison to the control. The MTT assay showed that Gd_2_O_3_-DEG nanoparticles are more toxic than other nanoparticles.

The MTT and LDH assay showed that Gd_2_O_3_-DEG nanoparticles are more toxic than Gd-DTPA and other nanoparticles. Also, Magnetoliposomes is more biocompatible than other nanoparticles. Magneto liposomes and Gd_2_O_3_-DEG nanoparticles, the two new synthesized and induced contrast agents are completely similar, except for their molecular weight. It appears that higher molecular weight can lead to more coverage and more biocompatibility.

Interestingly, they found no cellular damage evidence for 15-nm gold nanospheres bearing the same surface group. This result highlights the possible size-dependent toxicity of gold nanoparticles (30). In particular, gold nanoparticles less than 2 nm in diameter show chemical reactivity evidence that does not occur at larger sizes (31).

The conflicting results could arise from the variability of the used toxicity assays, cell lines, and nanoparticles chemical/physical properties. For example, cytotoxicity results can vary by using cell line. Citrate-capped gold nanoparticles (13 nm in diameter) were found to be toxic to a human carcinoma lung cell line, but not to human liver carcinoma cell line at the same dosage (32). Furthermore, varying the dose range parameters and the exposure time of gold nanoparticles to the cells in these studies, make it difficult to compare.

**Table 1:** DLS size and PdI measurement for two nanoparticle contrast agents. The results show a direct relationship between size and molecular weight

**Nanoparticles Hydrodynamic Diameter (nm)**		**PdI**
Gd2O3-DEG 27.29 ± 6.10		0.357
PMLNs 128.86 ± 36.24		0.387

We introduced two new nanoparticles with similar core and different coatings and assayed their toxicity in comparison with Gd-DTPA. In this study, Gd_2_O_3_ based contrast agents coated with two non-toxic different coatings. Therefore, among different groups’ coating materials, DEG, and liposomes due to their considerable properties containing different sizes and molecular weights, noticeable relaxivity, magnetic property, and high signal intensity, those are proper for cellular and molecular MRI applications that would be remained for future *in-vivo* studies.
